# Breast Tumor Classification in Ultrasound Images Using Combined Deep and Handcrafted Features

**DOI:** 10.3390/s20236838

**Published:** 2020-11-30

**Authors:** Mohammad I. Daoud, Samir Abdel-Rahman, Tariq M. Bdair, Mahasen S. Al-Najar, Feras H. Al-Hawari, Rami Alazrai

**Affiliations:** 1 Department of Computer Engineering, German Jordanian University, Amman 11180, Jordan; s.abdelRahman@gju.edu.jo (S.A.-R.); firas.alhawari@gju.edu.jo (F.H.A.-H.); rami.azrai@gju.edu.jo (R.A.); 2 Chair for Computer Aided Medical Procedure, Technical University of Munich, 85748 Munich, Germany; t.bdair@tum.de; 3 Department of Diagnostic Radiology, The University of Jordan Hospital, Queen Rania Street, Amman 11942, Jordan; m.najar@ju.edu.jo

**Keywords:** breast cancer, cancer detection, computer-aided diagnosis, tumor classification, deep learning, convolution neural networks, deep features, texture features, morphological features

## Abstract

This study aims to enable effective breast ultrasound image classification by combining deep features with conventional handcrafted features to classify the tumors. In particular, the deep features are extracted from a pre-trained convolutional neural network model, namely the VGG19 model, at six different extraction levels. The deep features extracted at each level are analyzed using a features selection algorithm to identify the deep feature combination that achieves the highest classification performance. Furthermore, the extracted deep features are combined with handcrafted texture and morphological features and processed using features selection to investigate the possibility of improving the classification performance. The cross-validation analysis, which is performed using 380 breast ultrasound images, shows that the best combination of deep features is obtained using a feature set, denoted by CONV features that include convolution features extracted from all convolution blocks of the VGG19 model. In particular, the CONV features achieved mean accuracy, sensitivity, and specificity values of 94.2%, 93.3%, and 94.9%, respectively. The analysis also shows that the performance of the CONV features degrades substantially when the features selection algorithm is not applied. The classification performance of the CONV features is improved by combining these features with handcrafted morphological features to achieve mean accuracy, sensitivity, and specificity values of 96.1%, 95.7%, and 96.3%, respectively. Furthermore, the cross-validation analysis demonstrates that the CONV features and the combined CONV and morphological features outperform the handcrafted texture and morphological features as well as the fine-tuned VGG19 model. The generalization performance of the CONV features and the combined CONV and morphological features is demonstrated by performing the training using the 380 breast ultrasound images and the testing using another dataset that includes 163 images. The results suggest that the combined CONV and morphological features can achieve effective breast ultrasound image classifications that increase the capability of detecting malignant tumors and reduce the potential of misclassifying benign tumors.

## 1. Introduction

Breast cancer is the most common cancer in females and a major cause of cancer-related deaths in women worldwide [1]. Ultrasound imaging is one of the widely used modalities for breast cancer diagnosis [2,3]. However, breast ultrasound (BUS) imaging is considered operator-dependent, and hence the reading of BUS images is a subjective task that requires well-trained and experienced radiologists [3,4]. Several studies, such as [5,6,7,8,9], proposed computer-aided diagnosis (CAD) systems to analyze BUS images with the goal of achieving objective computer-based classifications of breast tumors. In general, conventional CAD systems employ handcrafted features, such as the morphological features [10,11,12,13] and texture features [11,14,15], to characterize the tumor. Hence, the performance of conventional CAD systems depends mainly on the capability of the handcrafted features to capture the differences between benign and malignant tumors [16].

Several recent studies investigated the possibility of employing deep learning technology, and particularly convolutional neural networks (CNNs), to improve the performance of CAD systems [17,18]. However, a major challenge that restricts the full exploitation of CNNs for developing accurate CAD systems is the limited size of publicly available, well-annotated BUS image datasets that are crucial to achieve effective learning process [17,19]. Hence, the development of CNN models using limited BUS image datasets has become an important research challenge that was investigated in many previous studies [8,9,16,20,21,22,23,24,25]. A large group of these studies were essentially focused on the use of pre-trained CNNs to classify BUS images using two main strategies, namely the fine-tuning strategy and the deep features extraction strategy. In the fine-tuning strategy, the last fully connected layer of the pre-trained CNN model is modified to match the classes targeted by the BUS image classification problem and the parameters of the modified pre-trained CNN model are fine-tuned using the available BUS images. An example of this strategy is the study by Han et al. [9] who employed a modified GoogLeNet CNN model [26] that is pre-trained on the ImageNet dataset and fine-tuned using a BUS image dataset to classify the BUS images with a classification accuracy of 90%. In the study by Xiao et al. [23], three ImageNet pre-trained CNN models, namely Xception [27], InceptionV3 [28], and ResNet50 [29], were fine-tuned using a BUS image dataset to enable the classification of BUS images. Moreover, the classification performance achieved using these three fine-tuned CNN models was compared with the classification performance obtained using a dedicated CNN model that was developed specifically for the BUS image classification problem and trained using the same BUS image dataset. The results reported in [23] showed that the fine-tuned InceptionV3 CNN model achieved the highest classification performance with accuracy value of 85%. Tanaka et al. [24] compared the classification performance obtained using the ImageNet pre-trained VGG19 [30] and ResNet152 [29] CNN models, which were fine-tuned using a BUS image dataset, with the performance achieved by combining these two fine-tuned CNN models. The process of combining the two fine-tuned CNN models is achieved by averaging the class likelihoods achieved individually by each model. The results provided in [24] showed that the combined VGG19 and ResNet152 models achieved classification accuracy of 89%, which is higher than the accuracy obtained using each model alone. Despite the effective classification performance obtained using the fine-tuning strategy, this strategy involves several challenges, such as the selection of the parameters of the pre-trained model that are included in the fine-tuning process, configuring the learning rates, and ensuring that the available BUS images are sufficient to perform the fine-tuning process.

The second strategy, which involves the use of pre-trained CNNs to extract deep features from the BUS images and classify the extracted features using a computer classifier, was studied by several research groups. For instance, Byra et al. [21] employed the individual convolution layers of the ImageNet pre-trained VGG19 CNN model to extract deep features from the BUS image. The deep features were analyzed using Fisher linear discriminant analysis [31] to classify the BUS images with accuracy values as high as 80%. Antropova et al. [25] used the max-pooling layers and the first fully connected layer of the ImageNet pre-trained VGG19 CNN model to extract deep features for quantifying the BUS images. The extracted deep features were classified using a support vector machine (SVM) classifier [32]. The results reported in [25] showed that the deep features extracted from the max pooling layers were able to achieve the highest classification performance with area under the curve (AUC) of 0.87. In a recent study, Byra et al. [22] worked to improve the method proposed in [25] by adding a matching layer to interface the gray-scale BUS image with the three channels of the input layer of the ImageNet pre-trained VGG19 CNN model. Moreover, they compared the classification performance obtained by using the pre-trained VGG19 CNN model as a deep features extractor with the performance achieved by fine-tuning the pre-trained VGG19 model using a dataset of BUS images. The results reported in [22] showed that the matching layer was able to improve the BUS image classification performance. Furthermore, the results provided in [22] indicated that fine-tuning the pre-trained VGG19 CNN model using the BUS image dataset obtained the highest performance with classification accuracy of 88.7%, which is higher than the accuracies achieved by using the pre-trained VGG19 CNN model as a deep features extractor. A major drawback in the previous studies that employed the deep features extraction strategy is that the extracted deep features were not processed using feature selection algorithms to select the deep feature combinations that optimize the classification performance. In fact, the use of features selection algorithms is crucial to identify the most effective features and eliminate irrelevant features that might degrade the classification performance.

The main goal of the current study is to improve the classification performance obtained using the deep features extraction strategy. In addition, the current study aims to investigate the possibility of combining the deep features with conventional handcrafted features to achieve higher classification performance. The pre-trained CNN model considered in the current study for extracting the deep features is the ImageNet pre-trained VGG19 model since this model was commonly used in several previous studies [21,22,25] that employed the deep features extraction strategy. In fact, the main contributions of our study can be summarized as follows:

- Employ a two-phase optimization procedure to select the best deep feature combination that maximizes the BUS image classification performance as well as identify the components of the pre-trained VGG19 model that correspond to the optimized deep feature combination. In the first phase, we extracted deep features from the pre-trained VGG19 model at six different extraction levels. In the second phase, the deep features extracted at each extraction level are processed using a features selection algorithm and classified using a SVM classifier to identify the feature combinations that maximize the BUS image classification performance at that level. Furthermore, the deep feature combinations that are identified at all extraction levels are analyzed to find the best deep feature combination that enables the highest classification performance across all levels.
- Investigate the possibility of improving the BUS image classification performance by combining the deep features extracted from the pre-trained VGG19 model with the handcrafted features that were introduced in previous studies. In fact, the handcrafted features considered in the current study are the texture and morphological features. The features selection algorithm is used to select the best combination of deep and handcrafted features that maximizes the classification performance.
- Perform cross-validation analysis using a dataset that includes 380 BUS images to evaluate the classification performance obtained using the optimized combinations of deep features and combined deep and handcrafted features. Moreover, the classification performance obtained using the optimized combinations of deep features and combined deep and handcrafted features was compared with the results achieved using the optimized combinations of handcrafted features as well as the fine-tuned VGG19 model. Furthermore, the cross-validation analysis investigates the effect of classifying the deep features without applying the features selection algorithm.
- Evaluate the generalization performance of the optimized combination of deep features and the optimized combination of combined deep and handcrafted features. In particular, the 380 breast ultrasound images were used to train two SVM classifiers that employ the optimized combination of deep features and the optimized combination of combined deep and handcrafted features. The performance of the trained classifiers were evaluated using another dataset that includes 163 BUS images.

The remainder of the paper is organized as follows. Section 2 presents the BUS image datasets that are employed in the current study, the two-phase optimization procedure that is used to selected the best combination of deep features, and the handcrafted texture and morphological features that were combined with the deep features. Moreover, Section 2 introduces the procedures employed to evaluate the performance of the deep features, the combined deep features and handcrafted texture and morphological features, the handcrafted texture and morphological features, and the fine-tuned VGG19 model. The results are provided and discussed in Section 3. Finally, the conclusions are presented in Section 4.

## 2. Materials and Methods

### 2.1. BUS Image Datasets

Two BUS image datasets were employed in the current study. The first dataset, denoted by Dataset 1, was collected during the period between 2015 and 2019 at the Jordan University Hospital, Amman, Jordan. The dataset is composed of 380 BUS images (217 benign tumors and 163 malignant tumors). BUS image acquisition was carried out during routine breast cancer screening procedures using an Acuson S2000 system (Siemens AG, Munich, Germany) equipped with a 14L5 linear ultrasound transducer that has a bandwidth of 5–14 MHz. During imaging, the radiologist was free to adjust the parameters of the ultrasound imaging system, including the gain, depth, and focal length, to achieve the best view of the tumor. All acquired BUS images were resampled to have a uniform resolution of 0.1 mm × 0.1 mm. The tumors were labeled as benign or malignant based on biopsy findings. The mean ± standard deviation diameter of the tumors is 14.5 ± 5.8 mm. All participating patients were females. The mean ± standard deviation age of the patients was 47.8 ± 11.9 years. For each patient, one BUS image was acquired. The tumor in each BUS image was outlined by asking a radiologist (fourth author) with more than 15 years of experience to segment the tumor for three times and the gold standard segmentation was taken as the mean of the three manual segmentations. The study protocol was approved by the Ethics Committee at the Jordan University Hospital. Furthermore, each participating patient was asked to sign informed consent to the study protocol.

The second BUS dataset, denoted by Dataset 2, was provided by the authors of [33]. The dataset was acquired in 2012 at the UDIAT Diagnostic Centre of the Parc Tauli Corporation, Sabadell, Spain. The dataset is composed of 163 BUS images (110 benign tumors and 53 malignant tumors). As described in [33], the tumors in the BUS images were outlined by experienced radiologists. Detailed description of the dataset is provided in [33].

### 2.2. BUS Image Classification Using the Deep Features

A two-phase procedure is used to identify the best combination of deep features, which are extracted using the ImageNet pre-trained VGG19 model that maximizes the BUS image classification performance as well as the components of the pre-trained VGG19 model that correspond to the best deep feature combination. This two-phase procedure is applied using the BUS images included in Dataset 1. The first phase of this procedure, which is described in Section 2.2.1, aims to extract deep features from the pre-trained VGG19 model at six different extraction levels. The second phase, which is described in Section 2.2.2, aims to process the deep features that are extracted at each level using features selection and classification to identify the feature combinations that maximize the classification performance at that extraction level. The deep feature combination that achieves the highest classification performance across all six deep features extraction levels is denoted as the best-performing combination of deep features.

#### 2.2.1. Deep Features Extraction

**Figure 1:**
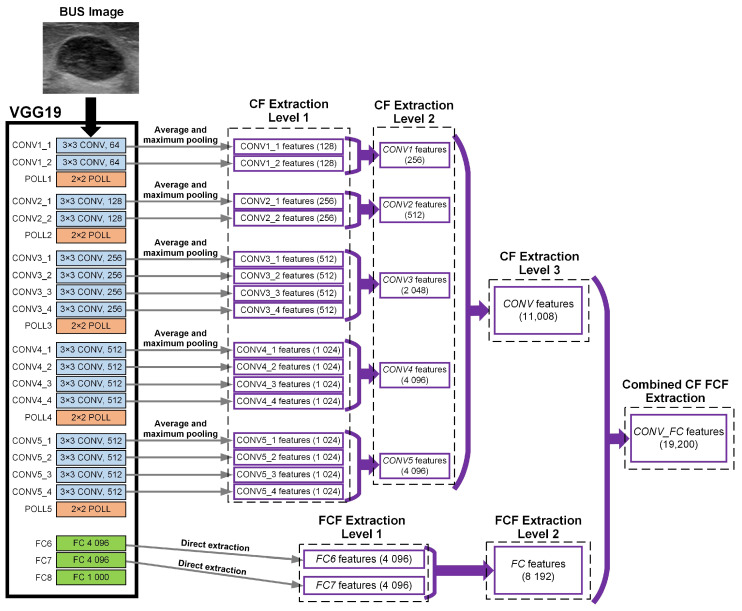
Graphical illustration of the process of extracting deep features from the ROI in the BUS image using the pre-trained VGG19 model.

Each BUS image in Dataset 1 is processed to define a region of interest (ROI) around the tumor. As suggested in [22], the ROI is obtained by computing the smallest box that contains the tumor and adding 30 pixels to each side of the box to include the surrounding tissue. For each image, the pre-trained VGG19 model [30] is used to extract deep features from the ROI that includes the tumor, as illustrated in Figure 1. As shown in the figure, the VGG19 model is composed of five convolution blocks, where each block includes a group of convolution layers followed by a max-pooling layer, and three fully connected layers. The first two convolution blocks comprise two convolution layers that use 64 kernels with a size of 3×3. The third convolution block is composed of four convolution layers that employ 256 kernels with a size of 3×3. The last two convolution blocks include four convolution layers that use 512 kernels with a size of 3×3. The first two fully connected layers (FC6 and FC7) have 4096 units and the last fully connected layer (FC8) includes 1000 units that correspond to the 1000 classes of the ImageNet dataset [34].

The deep features extracted using the VGG19 model can be grouped into two main categories: convolution features and fully connected features. The convolution features are extracted from the convolution layers based on the method described in [35]. In particular, consider a convolution layer, denoted by CL, with a size of W×H×N, where *W*, *H*, and *N* are the width, height, and number of convolution kernels of the layer. The convolution layer, CL, can be processed to extract *N* convolution features, denoted by $CL_{max}$, using the maximum pooling operation as follows [35]:

(1) $$\begin{array}{c} CL_{max}=\left[CL_{max}\left(1\right),CL_{max}\left(2\right),...,CL_{max}\left(N\right)\right],\;\;\;\mathrm{where}\\ CL_{max}\left(k\right)=max\left\{CL(.,.,k)\right\},\;\;\;k=1,2,3,...,N\;\;\;\;\;\;\;\;\;\;\;\;\; \end{array}$$

In addition, the CL can be processed to extract *N* convolution features, denoted by $CL_{avg}$, using the average pooling operation, as expressed below [35]:

(2) $$\begin{array}{c} CL_{avg}=\left[CL_{avg}\left(1\right),CL_{avg}\left(2\right),...,CL_{avg}\left(N\right)\right],\;\;\;\mathrm{where}\\ CL_{avg}\left(k\right)=\frac{1}{W\times H}\sum CL\left(.,.,k\right),\;\;\;k=1,2,3,...,N\;\;\; \end{array}$$

In the current study, a three-level convolution features extraction approach is employed. In the first level, denoted as convolution features extraction level 1 (CF Extraction Level 1), the convolution features, i.e., $CL_{avg}$ and $CL_{avg}$, extracted from each convolution layer are concatenated to form a feature set with a size of 2N and this set is normalized using $l_{2}$-normalization [36]. As illustrated in Figure 1, eighteen feature sets were extracted at CF Extraction Level 1, where each set includes between 128 and 1024 convolution features. In the second level, denoted as convolution features extraction level 2 (CF Extraction Level 2), the feature sets extracted from all layers of each convolution block of the VGG19 model are concatenated to form a feature set and this set is normalized using $l_{2}$-normalization. As shown in Figure 1, five feature sets were extracted at CF Extraction Level 2, where each set includes between 256 and 4096 convolution features. In the third level, called convolution features extraction level 3 (CF Extraction Level 3), the feature sets extracted from all convolution blocks of the VGG19 model are concatenated to form a feature set and this set is normalized using $l_{2}$-normalization. As shown in Figure 1, the feature set extracted at CF Extraction Level 3 is composed of 11,008 convolution features.

The VGG19 model is also used to extract fully connected features from the ROI that includes the tumor. Particularity, a two-level fully connected features extraction approach is employed. In the first level, denoted as fully connected features extraction level 1 (FCF Extraction Level 1), the activations of each fully connected layer are extracted and normalized using $l_{2}$-normalization to form a feature set. As shown in Figure 1, two fully connected feature sets were extracted at FCF Extraction Level 1, where each set includes 4096 fully connected features. In the second level, called fully connected features extraction level 2 (FCF Extraction Level 2), the two feature sets extracted at FCF Extraction Level 1 are concatenated and normalized using $l_{2}$-normalization to form one feature set. As shown in Figure 1, the feature set extracted at FCF Extraction Level 2 is composed of 8192 fully connected features.

In addition to the five deep features extraction levels described above, an additional deep features extraction level, called combined convolution and fully connected features extraction (Combined CF FCF Extraction), is used to extract deep features from all convolution blocks and all fully connected layers of the VGG19 model. The features extracted at Combined CF FCF Extraction are concatenated and normalized using $l_{2}$-normalization to form one feature set. As shown in Figure 1, the feature set computed at Combined CF FCF Extraction includes 19,200 convolution and fully connected features. The six deep features extraction levels that are employed in the current study are summarized in Table 1.

#### 2.2.2. Deep Features Selection and Classification

The use of the extracted features to directly classify the BUS images in Dataset 1 can limit the classification performance as these features might include redundant and irrelevant information [37]. To overcome this limitation, the extracted features are processed using features selection to identify the relevant and effective features that can achieve high classification performance. Despite the fact that the exhaustive search of all possible feature combinations can identify the optimal feature combination, this search approach requires long processing times and extensive computational resources, particularly when a large number of features is considered [37]. In the current study, features selection is performed using a two-phase heuristic procedure [7] that is based on the features selection algorithm described in [14]. In the first phase, the extracted features are ranked using the minimum redundancy maximum relevance (mRMR) features selection algorithm [38]. After ranking the features, *M* feature groups are formed such that the mth feature group is composed of the *m* top-ranked features, where *M* is the total number of features and m={1,2,3,…,M}. Moreover, the classification performance of the *M* feature groups is evaluated using the Matthews correlation coefficient (MCC) metric [39]. The MCC is used as it provides an effective classification performance metric for imbalanced data [40]. Then, the smallest feature group that achieves the highest classification performance is selected as the candidate feature group. In the second phase, the candidate feature group obtained by the first phase is refined using a backward elimination procedure to achieve a compact subset of features that maximizes the classification performance. Assume that the size of the candidate feature group obtained by the first phase is equal to *n*, then the aim of the first iteration of the backward elimination procedure is to identify a subset of n−1 features that can achieve the highest improvement in classification performance. In particular, each individual feature in the candidate feature group, which includes *n* features, is temporally eliminated and the classification performance obtained by the remaining n−1 features is evaluated. The feature that its elimination leads to the highest improvement in the classification performance is identified and permanently removed to obtain a subset of n−1 features with enhanced classification performance. This process is repetitively applied in the subsequent iterations of the backward elimination procedure to reduce the size of the selected features and, at the same time, improve the classification performance. This iterative process stops when the elimination of any feature leads to a reduction in the classification performance.

The process of classifying the features is performed using a binary SVM classifier [32] that is implemented using the LIBSVM library [41]. The SVM classifier is configured to use the radial basis function (RBF) kernel. Using this configuration, the SVM classifier has two control parameters that require tuning, namely the regularization parameter (*C*) and the RBF kernel parameter (σ). The training and testing of the SVM classifier is performed using a ten-fold cross-validation procedure combined with a grid search approach to tune the parameters *C* and σ. The ten-fold cross-validation procedure is used to reduce the possibility of overfitting the SVM classifier to the 380 BUS images included in Dataset 1 [42]. To carry out the ten-fold cross-validation procedure, the BUS images in Dataset 1 are divided into ten groups such that each group includes 38 BUS images. In each iteration of the ten-fold cross-validation procedure, nine groups of BUS images are used to train the SVM classifier and the remaining group is used for testing. This train-test process is repeated for ten iterations such that each group of BUS images is used exactly once as a testing group. Moreover, as suggested in [43,44], the tuning of *C* and σ is carried out using a grid search approach that examines the *C* and σ values of $\left\{2^{-5},2^{-4},2^{-3},\ldots ,2^{15}\right\}$ and $\left\{2^{-15},2^{-14},2^{-13},\ldots ,2^{3}\right\}$, respectively. The grid search approach is configured to find the values of *C* and σ that maximize the classification performance that is evaluated using the MCC metric. In fact, the MCC is employed since it provides an effective classification metric for imbalanced data, where such data imbalance can occur due to the unequal numbers of benign and malignant BUS images [40].

### 2.3. BUS Image Classification by Combining the Deep Features with Handcrafted Features

We investigated the possibility of improving the classification of the BUS images included in Dataset 1 by combining the deep features with handcrafted features that were introduced in previous studies. In particular, the best-performing deep feature set extracted from the pre-trained VGG19 model is combined with handcrafted texture and morphological features that are commonly used for BUS image classification. To extract the handcrafted texture features, the ROI in the BUS image that includes the tumor is analyzed using the Gray-Level Co-occurrence Matrix (GLCM) [45] to quantify the statistics of the pixels’ intensities within the ROI. In the current study, 10 distances (d=1,2,3,…,10pixels) and 4 orientations $\left(\theta =0^{\circ },45^{\circ },90^{\circ },135^{\circ }\right)$ are employed to compute the GLCM, as suggested in [14]. This process enabled the creation of 40 GLCMs. Each GLCM is analyzed to extract 20 texture features that are widely employed for BUS image classification [7,14,46]. These texture features are summarized in Table 2. Hence, the total number of texture features that are computed for each BUS image is equal to 800.

In addition, the tumor outline in each BUS image is analyzed to extract 18 handcrafted morphological features, which are summarized in Table 2. In particular, 10 morphological features, namely the tumor area [12], tumor perimeter [12], tumor form factor [10,13], tumor roundness [10,13], tumor aspect ratio [10,13], tumor convexity [10,13], tumor solidity [10,13], tumor extent [10,13], tumor undulation characteristics [47], and tumor compactness [12,48], are extracted directly from the tumor outline. Two morphological features are extracted by computing the the normalized radial length (NRL) of the tumor and calculating its entropy and variance [12,49]. In fact, the NRL of the tumor is the Euclidean distance between the tumor center and the pixels located at the tumor boundary normalized by the maximum distance [49]. The last 6 morphological features are extracted from the best-fit ellipse that is obtained by fitting an ellipse to the tumor outline [12]. These 6 features are the length of the ellipse major axis [12], the length of the ellipse minor axis [12], the ratio between the ellipse major and minor axes [12], the ratio of the ellipse perimeter and the tumor perimeter [12], the angle of the ellipse major axis [12], and the overlap between the ellipse and the tumor [12].

The best-performing deep feature set, the handcrafted texture features, and the handcrafted morphological features are concatenated to form three feature groups that combine deep and handcrafted features. The first group comprises the best-performing deep feature set and the handcrafted texture features. The second group comprises the best-performing deep feature set and the handcrafted morphological features. Finally, the third group is composed of the best-performing deep feature set and the handcrafted texture and morphological features. Each one of these feature groups is normalized using $l_{2}$-normalization and processed using features selection and classification to find the best combination of deep and handcrafted features that optimizes the classification of the BUS images included in Dataset 1.

### 2.4. Performance Comparison

For the BUS images included in Dataset 1, we compared the classifications obtained using the deep feature sets and the groups of combined deep and handcrafted features with the classifications achieved using three other classification approaches. The first approach aims to investigate the importance of the features selection algorithm, which eliminates the irrelevant and redundant features. In particular, the first approach considers the BUS image classifications that are obtained using the best-performing deep feature set but without applying the features selection algorithm. In other words, the first approach is focused on the BUS image classifications that are achieved by applying all features included in the best-performing deep feature set directly to the SVM classifier. The second approach aims to evaluate the classification performance that can be achieved using the handcrafted texture and morphological features. In particular, the second approach considers the BUS image classifications obtained using the 800 handcrafted texture features, the 18 handcrafted morphological features, and the combined 818 handcrafted texture and morphological features, which are described in Section 2.3, after applying features selection. The third approach considers the BUS image classification performance achieved by fine-tuning the pre-trained VGG19 model using the BUS images included in Dataset 1. The process of fine-tuning the pre-trained VGG19 model is performed using the fine-tuning procedure presented in [22].

### 2.5. Generalization Performance

The generalization performance of the deep feature sets and the groups of combined deep and handcrafted features was investigated in the current study. In particular, the BUS images in Dataset 1 are used to train two SVM classifiers, where the first classifier is based on the best-performing deep feature set and the second classifier is based on the best-performing group of combined deep and handcrafted features. In fact, the best-performing deep feature set and the best-performing group of combined deep and handcrafted features are identified based on the ten-fold cross-validation analyses performed for Database 1, as described in Section 2.2 and Section 2.3, respectively. The generalization performance of the two SVM classifiers was evaluated by employing each classifier to classify the 163 BUS images included in Dataset 2.

### 2.6. Performance Metrics

Six performance metrics were used to evaluate the cross-validation classifications performed for the BUS images in Dataset 1 based on the deep feature sets, the groups of combined deep and handcrafted features, and the three classification approaches used in the performance comparison. These metrics are the accuracy, sensitivity, specificity, Matthews correlation coefficient (MCC), positive predictive value (PPV), and negative predictive value (NPV). The mathematical formulas of these metrics are provided below [39,52]:

(3) $$\begin{array}{c} Accuracy=\frac{TP+TN}{TP+TN+FP+FN}\;,\\ Sensitivity=\frac{TP}{TP+FN}\;,\\ Specificity=\frac{TN}{TN+FP}\;,\\ MCC=\frac{TP\times TN-FP\times FN}{\sqrt{\left(TP+FP)(TP+FN)(TN+FP)(TN+FN)\right)}}\;,\\ PPV=\frac{TP}{TP+FP}\;,\\ NPV=\frac{TN}{TN+FN}\;,\; \end{array}$$

where *TP* is the number of malignant tumors that are classified correctly, *TN* is the number of benign tumors that are classified correctly, *FP* is the number of benign tumors that are classified incorrectly, and FN is the number of malignant tumors that are classified incorrectly. In fact, the six performance metrics were computed for each one of the ten folds that carried out during the cross-validation analysis performed for Dataset 1. Moreover, the mean ± standard deviation values of the six metrics are calculated across the ten folds.

In addition to the six performance metrics, the receiver operator characteristic (ROC) curves were computed for the cross-validation classifications performed for Dataset 1 using the best-performing combination of deep features, the best-performing combination of deep and handcrafted features, the best-performing combination of handcrafted features, and the fine-tuned VGG19 model. In fact, the ROC curves aim to study the relation between the classification sensitivity and specificity obtained using each one of these classification approaches. The values of the AUC, which evaluates the overall performance of the BUS image classifier, were also calculated for these four classification approaches. In addition to the ROC curve analysis, these four classification approaches were studied using paired *t*-tests based on the BUS image classification accuracies. In fact, the aim of the paired *t*-tests is to investigate if the BUS image classification accuracy obtained using the most powerful approach, out of the four approaches described above, is significantly different than the other three approaches.

The generalization performance of the best-performing deep feature set and the best-performing group of combined deep and handcrafted features, which are achieved by performing the training using Dataset 1 and the testing using Dataset 2, was evaluated using the six metrics described above. In particular, the values these metrics were computed based on the classifications obtained for the 163 BUS images included in Dataset 2.

## 3. Results and Discussions

### 3.1. BUS Image Classification Results Obtained Using the Deep Features

The classification results obtained using the ten-fold cross-validation analyses that are performed for the BUS images in Dataset 1 based on the deep feature sets are provided in Table 3. The highest classification performance is achieved using the CONV feature set that is extracted at CF Extraction Level 3. The CONV feature set achieved BUS image classification with mean accuracy of 94.2%, mean sensitivity of 93.3%, mean specificity of 94.9%, mean PPV of 93.3%, mean NPV of 94.9%, and mean MCC of 88.2%. In fact, the features selection procedure was able to process the 11,008 features included in the CONV feature set to select a combination of 25 features that achieved the highest classification performance. These 25 features are extracted from six convolution layers of the pre-trained VGG19 model, which are CONV3_3 (1 feature), CONV4_2 (3 features), CONV4_3 (1 feature), CONV4_4 (1 feature), CONV5_1 (5 features), and CONV5_4 (14 features). Furthermore, Table 3 indicates that the classification results achieved using the CONV_FC feature set matches the classification results obtained using the CONV feature set. This is due to the fact that the feature combination selected for the CONV_FC feature set matches the feature combination selected for the CONV feature set. Hence, the fully connected features included in the CONV_FC feature set were unable to improve the classification performance achieved by the convolution features included in both the CONV_FC feature set and the CONV feature set. Hence, the best-performing deep feature set that is extracted from the pre-trained VGG19 model is considered to be the CONV feature set.

In addition, the results provided in Table 3 show that the classification results achieved using the feature sets extracted at CF Extraction Level 2 are generally higher than the results obtained using the feature sets extracted at CF Extraction Level 1. This can be attributed to the fact that each feature set extracted at CF Extraction Level 2 is composed of the features obtained from a particular convolution block of the VGG19 model, while the feature sets extracted at CF Extraction Level 1 is composed of the features obtained from the individual convolution layers of the VGG19 model. Hence, for the feature sets extracted at CF Extraction Level 2, the features selection procedure can process the features extracted from all layers of each convolution block of the VGG19 model. However, in the case of the feature sets extracted at CF Extraction Level 1, the search space of the features section procedure is limited to the features obtained from the individual convolution layers of the VGG19 model.

For CF Extraction Level 1, the results presented in Table 3 indicate that the feature sets associated with high convolution layers, such as CONV5_4 and CONV5_3, generally achieved better classification results compared to the feature sets associated with low convolution layers, such as CONV1_1 and CONV1_2. Similarly, for CF Extraction Level 2, the feature sets associated with high convolution blocks, such as CONV5, obtained better classification results compared to the feature sets associated with low convolution blocks, such as CONV1. This can be attributed to the fact that high convolution layers tend to learn abstract features while low convolution layers extract low-level features, such as the edges [53].

### 3.2. BUS Image Classification Results Obtained by Combining the Deep Features with Handcrafted Features

Table 4 provides a comparison between the classification results achieved by the CONV feature set and the results obtained by combining the CONV feature set with the handcrafted texture, morphological, and combined texture and morphological features. In fact, this comparison is performed using the ten-fold cross-validation procedure that is applied on the BUS images in Dataset 1. As shown in Table 4, the highest classification performance is achieved by combining the CONV feature set (11,008 features) with the handcrafted morphological features (18 features) and processing these combined features using features selection to select a combination that consists of 18 CONV features and 3 morphological features. In fact, the selected combination of CONV and morphological features enabled the classification of BUS images with mean accuracy of 96.1%, mean sensitivity of 95.7%, mean specificity of 96.3%, mean PPV of 95.1%, mean NPV of 96.8%, and mean MCC of 92.0%. These results indicate that the morphological features, which quantify the shape and structure of the tumor, include tumor quantification information that complement the tumor quantifications that are achieved by the CONV feature set. Hence, the combined CONV feature set and morphological features were able to achieve higher classification performance compared with the CONV feature set alone. The high classification sensitivity obtained by combining the CONV feature set with the morphological features indicates that these combined features enable high detection capability of malignant breast tumors. In addition, the high classification specificity achieved by the combined CONV feature set and morphological features indicate that these combined features reduce the potential of misclassifying benign tumors, which in turn reduces the number of unnecessary biopsies performed for benign tumors.

Furthermore, the results provided in Table 4 indicate that the classification performance achieved by the CONV feature set matches the classification performance obtained by combining the CONV feature set with the handcrafted texture features. In addition, the classification performance achieved by combining the CONV feature set with the handcrafted morphological features matches the classification performance obtained by combining the CONV feature set with the handcrafted morphological and texture features. This is attributed to the fact that the features selected by the features selection algorithm for the CONV feature set are the same features that are selected for the combined CONV feature set and texture features. In addition, the features selected for the combined CONV feature set and morphological features are the same features that are selected for the combined CONV feature set, morphological features, and textures features. These findings suggest that the tumor quantifications obtained by the CONV feature set include the tumor quantifications achieved by the handcrafted texture features.

### 3.3. Performance Comparison Results

Table 5 provides a comparison between the results obtained using the CONV feature set with and without features selection, the combined CONV feature set and handcrafted morphological features with features selection, the handcrafted texture, morphological, and combined texture and morphological features with features selection, and the fine-tuned VGG19 model. In fact, this comparison is performed using the ten-fold cross-validation procedure that is applied on the BUS images in Dataset 1. As shown in the table, the highest classification performance is achieved using the combined CONV feature set and morphological features after applying the features selection algorithm. Moreover, the second highest classification performance is obtained using the CONV feature set after applying the features selection algorithm.

Furthermore, Table 5 shows that the classification results obtained using the CONV feature set degraded when the features selection algorithm is not applied. This is attributed to the fact that the CONV feature set is composed of 11,008 features that include relevant features, which enable effective BUS image classification, as well as redundant and irrelevant features, which degrade the classification performance. Hence, the performance of the SVM classifier degraded significantly when all 11,800 features are classified directly by the classier. On the other hand, when the CONV feature set is processed using the features selection algorithm, the most effective features are selected and used to classify the BUS images.

In addition, Table 5 shows that the handcrafted texture, morphological, and combined texture and morphological features, which are processed using the features selection algorithm, achieved low classification performance compared to the CONV feature set and the combined CONV feature set and morphological features when the features selection algorithm is applied. In addition, Table 5 indicates that the classification performance obtained using the morphological features is higher than the texture features. Furthermore, the classification performance is improved by combining the texture and morphological features. These findings agree with the BUS image classification results reported in previous studies, such as [7]. Furthermore, Table 5 indicates that the fine-tuned VGG19 model achieved classification results that are lower than the results obtained using the CONV feature set when the features selection algorithm is applied. On the other hand, the fine-tuned VGG19 model outperformed the CONV feature set when the features selection algorithm is not applied.

**Figure 2:**
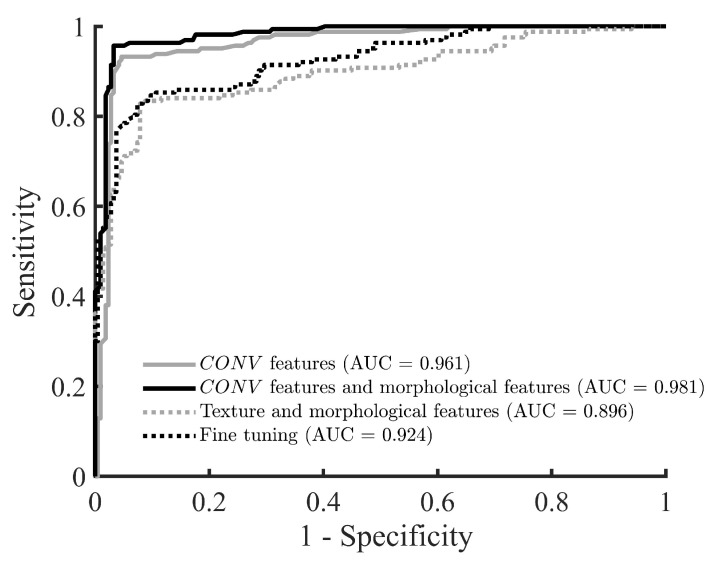
The ROC curves obtained using the CONV feature set (with features selection), the combined CONV feature set and morphological features (with features selection), the combined handcrafted texture and morphological features (with features selection), and the fine-tuned VGG19 CNN model.

Figure 2 shows the ROC curves obtained using the CONV feature set and the combined CONV feature set and morphological features after applying the features selection algorithm. Furthermore, the figure shows the ROC curve achieved using the combined texture and morphological features after applying the features selection algorithm as well as the ROC curve obtained using the fine-tuned VGG19 model. The highest AUC value is obtained using the combined CONV feature set and morphological features. Moreover, the second highest AUC value is achieved using the CONV feature set. These results confirm the superior classification performance achieved by combining the CONV feature set with the morphological features.

The *p* values obtained using the paired *t*-tests to compare the classification accuracies of the combined CONV feature set and morphological features after applying features selection with the CONV feature set after applying features selection, the combined texture and morphological features after applying features selection, and the fine-tuned VGG19 model are equal to 0.043, 0.003, and 0.001. These *p* values indicate that the classification accuracy achieved by the combined CONV feature set and morphological features is significantly different than the three other classification approaches at confidence level of 0.05.

### 3.4. Generalization Performance Results

For the CONV feature set, the values of the classification accuracy, sensitivity, specificity, PPV, NPV, and MCC obtained by performing the training using Dataset 1 and the testing using Dataset 2 are equal to 93.3%, 90.7%, 94.5%, 89.1%, 95.4%, and 84.8%, respectively. Furthermore, the values of the classification accuracy, sensitivity, specificity, PPV, NPV, and MCC achieved using the combined CONV feature set and morphological features are equal to 95.1%, 94.4%, 95.4%, 91.1%, 97.2%, and 89.1%, respectively. These results are close to the corresponding results in Table 4, which are obtained using the ten-fold cross-validation analysis that is applied on Dataset 1. This finding suggests that the classification results reported in the current study can be generalized to other BUS image datasets.

## 4. Conclusions

The current study contributes to the ongoing efforts to improve BUS image classification by extracting deep features from the pre-trained VGG19 model at six different deep features extraction levels, combining the extracted deep features with handcrafted texture and morphological features, processing the features using a features selection algorithm, and classifying the selected features using a SVM classifier. The results reported in the current study indicate that the highest classification performance that can be achieved using the deep features is enabled using the CONV feature set, which includes features extracted from the layers of all convolution blocks of the VGG19 model. In addition, the results show that the classification performance of the CONV feature set can be improved by combining this feature set with handcrafted morphological features. In particular, the combined CONV feature set and morphological features achieved mean accuracy of 96.1%, mean sensitivity of 95.7%, mean specificity of 96.3%, mean PPV of 95.1%, mean NPV of 96.8%, and mean MCC of 92.0%. On the other hand, combining the CONV feature set with the handcrafted texture features did not improve the classification performance, which suggests that the tumor quantifications provided by the handcrafted texture features are included in the CONV feature set. The high sensitivity and specificity values obtained by the combined CONV feature set and morphological features can enable high detection capability of malignant tumors and reduce the potential of misclassifying benign tumors as malignant tumors. Furthermore, the performance comparison results provided in the current study show that both the CONV feature set and the combined CONV feature set and morphological features outperform the handcrafted texture and morphological features and the fine-tuned VGG19 model. The results also show that the performance of the CONV feature set degrades when the features selection algorithm is not applied. This suggests the importance of processing the deep features using features selection algorithms to enable high classification performance. The generalization performance analysis conducted in the current study indicates that the CONV feature set and the combined CONV feature set and morphological features can be used to achieve high classification performance in other BUS image datasets. The future directions of the current study include combining the deep features that are extracted from the BUS image using different pre-trained CNN models with the goal of improving the classification performance.

## Figures and Tables

**Table 1:** The six levels that are employed to extract the deep feature sets from the BUS image.

Deep Features Extraction Level	Feature Sets	Description
CF Extraction Level 1	CONV1_1, CONV1_2, CONV2_1, CONV2_2, CONV3_1, CONV3_2, CONV3_3, CONV3_4, CONV4_1, CONV4_2, CONV4_3, CONV4_4, CONV5_1, CONV5_2, CONV5_3, CONV5_4	A total of 11,008 convolution features organized into 16 feature sets are extracted from the ROI that includes the tumor, where each feature set corresponds to one of the convolution layers of the VGG19 model. To generate a given feature set, the convolution features $CL_{avg}$ and $CL_{avg}$ extracted from the convolution layer that corresponds to the feature set are concatenated and normalized.
CF Extraction Level 2	CONV1, CONV2, CONV3, CONV4, CONV5	A total of 11,008 convolution features organized into 5 feature sets are extracted from the ROI that includes the tumor, where each feature set corresponds to one of the convolution blocks of the VGG19 model. To generate a given feature set, the feature sets extracted from the layers of the convolution block that corresponds to the feature set are concatenated and normalized.
CF Extraction Level 3	CONV	A total of 11,008 convolution features organized into 1 feature set are extracted from the ROI that includes the tumor. To generate the feature set, the feature sets extracted from all convolution blocks of the VGG19 model are concatenated and normalized.
FCF Extraction Level 1	FC6 and FC7	Two feature sets, where each set includes 4096 fully connected features, are extracted from the ROI that includes the tumor. The computation of the two feature sets is achieved by extracting and normalizing the activations of first and second fully connected layers of the VGG19 model.
FCF Extraction Level 2	FC	A feature set that includes 8192 fully connected features is extracted from the ROI that includes the tumor. The computation of the feature set is achieved by concatenating and normalizing the two feature sets extracted from the first and second fully connected layers of the VGG19 model.
Combined CF FCF Extraction	CONV_FC	A feature set that includes 19,200 convolution and fully connected features is extracted from the ROI that includes the tumor. The computation of the feature set is performed by extracting deep features from all convolution blocks and all fully connected layers of the VGG19 model and then concatenating and normalizing the extracted features.

**Table 2:** The handcrafted texture and morphological features that are extracted from the BUS image.

Type	Features	Description
Texture features	Autocorrelation [50], contrast [14], correlation [50], cluster prominence [50], cluster shade [50], dissimilarity [50], energy [50], entropy [50], homogeneity [50], maximum probability [50], sum of squares [45], sum average [45], sum entropy [45], sum variance [45], difference variance [45], difference entropy [45], information measure of correlation I [45], information measure of correlation II [45], inverse difference normalized [51], inverse difference moment normalized [51]	A total of 800 texture features are extracted from the ROI that includes the tumor. In particular, 40 GLCMs are generated using 10 distances (d=1,2,3,…,10pixels) and 4 orientations $\left(\theta =0^{\circ },45^{\circ },90^{\circ },135^{\circ }\right)$. Moreover, each GLCM is analyzed to extract 20 texture features.
Morphological features	Tumor area [12], tumor perimeter [12], tumor form factor [10,13], tumor roundness [10,13], tumor aspect ratio [10,13], tumor convexity [10,13], tumor solidity [10,13], tumor extent [10,13], tumor undulation characteristics [47], tumor compactness [12,48], NRL entropy [12,49], NRL variance [12,49], length of the ellipse major axis [12], length of the ellipse minor axis [12], ratio between the ellipse major and minor axes [12], ratio of the ellipse perimeter and the tumor perimeter [12], angle of the ellipse major axis [12], overlap between the ellipse and the tumor [12]	A total of 18 morphological features are extracted from the tumor outline. In particular, 10 morphological features are computed directly based on the tumor outline. Moreover, 2 morphological features are computed based on the NRL of the tumor. In addition, 6 morphological features are computed by fitting an ellipse to the tumor outline.

**Table 3:** The BUS image classification results obtained using the feature sets extracted from the pre-trained VGG19 model at six deep features extraction levels. For the six performance metrics, the mean ± standard deviation values are computed across the ten folds of the cross-validation procedure performed using Dataset 1.

Deep Features Extraction Level	Feature Set	Total No. of Features	No. of Selected Features	Selected Features	Accuracy (%)	Sensitivity (%)	Specificity (%)	PPV (%)	NPV (%)	MCC (%)
CF Extraction Level 1	CONV1_1	128	34	CONV1_1 (34)	78.2±6.9	76.7±7.4	79.3±11.8	73.5±12.0	81.9±6.8	55.7±12.7
	CONV1_2	128	21	CONV1_2 (21)	81.8±5.2	81.6±8.9	82.0±9.7	77.3±11.0	85.6±5.5	63.3±10.1
	CONV2_1	256	25	CONV2_1 (25)	82.1±5.2	77.3±9.3	85.7±8.9	80.3±8.9	83.4±6.2	63.3±9.8
	CONV2_2	256	38	CONV2_2 (38)	84.2±3.7	80.4±9.4	87.1±7.7	82.4±7.4	85.5±7.6	67.7±6.2
	CONV3_1	512	33	CONV3_1 (33)	84.7±4.8	80.4±11.9	88.0±4.4	83.4±2.1	85.7±8.1	68.7±9.2
	CONV3_2	512	47	CONV3_2 (47)	86.1±4.5	82.2±8.1	88.9±5.5	84.8±7.4	86.9±5.7	71.4±9.0
	CONV3_3	512	29	CONV3_3 (29)	85.3±4.5	79.8±10.5	89.4±5.7	85.0±6.2	85.5±7.0	69.8±8.1
	CONV3_4	512	86	CONV3_4 (86)	88.9±4.4	85.3±10.0	91.7±3.1	88.5±4.1	89.2±7.1	77.4±8.6
	CONV4_1	1024	20	CONV4_1 (20)	88.4±5.0	84.0±7.6	91.7±5.6	88.4±7.0	88.4±5.8	76.3±10.2
	CONV4_2	1024	61	CONV4_2 (61)	87.1±4.0	82.8±8.3	90.3±2.6	86.5±4.4	87.5±6.0	73.6±7.8
	CONV4_3	1024	31	CONV4_3 (31)	86.8±5.5	82.8±9.3	89.9±5.4	86.0±5.7	87.4±7.0	73.1±10.8
	CONV4_4	1024	32	CONV4_4 (32)	90.0±4.8	89.0±10.3	90.8±4.9	87.9±9.3	91.6±6.0	79.6±10.5
	CONV5_1	1024	58	CONV5_1 (58)	89.5±5.0	87.7±11.2	90.8±6.0	87.7±8.1	90.8±6.1	78.5±11.0
	CONV5_2	1024	41	CONV5_2 (41)	89.7±3.2	88.3±9.1	90.8±5.2	87.8±8.5	91.2±5.5	79.1±6.8
	CONV5_3	1024	23	CONV5_3 (23)	89.7±4.4	87.1±8.9	91.7±6.5	88.8±10.7	90.5±6.5	79.0±9.1
	CONV5_4	1024	31	CONV5_4 (31)	91.3±4.1	91.4±5.4	91.2±6.7	88.7±10.1	93.4±3.2	82.4±9.2
CF Extraction Level 2	CONV1	256	34	CONV1_1 (17), CONV1_2 (17)	82.6±5.1	82.8±7.3	82.5±7.5	78.0±9.2	86.5±5.5	64.9±10.0
	CONV2	512	23	CONV2_1 (5), CONV2_2 (18)	84.7±3.7	80.4±9.4	88.0±7.0	83.4±6.9	85.7±7.6	68.7±6.1
	CONV3	2048	15	CONV3_1 (7), CONV3_4 (8)	88.7±4.3	84.0±9.0	92.2±5.8	89.0±7.7	88.5±6.3	76.8±8.3
	CONV4	4096	34	CONV4_1 (8), CONV4_2 (4), CONV4_3 (8), CONV4_4 (14)	90.5±4.8	88.3±10.1	92.2±4.4	89.4±9.1	91.3±5.9	80.6±10.6
	CONV5	4096	27	CONV5_2 (7), CONV5_3 (8), CONV5_4 (12)	91.8±4.5	91.4±5.4	92.2±6.5	89.8±10.5	93.5±3.2	83.4±10.0
CF Extraction Level 3	**CONV**	11,008	25	CONV3_3 (1), CONV4_2 (3), CONV4_3 (1), CONV4_4 (1), CONV5_1 (5), CONV5_4 (14)	**94.2** ± **2.7**	**93.3** ± **5.1**	**94.9** ± **4.1**	**93.3** ± **5.6**	**94.9** ± **4.4**	**88.2** ± **5.5**
FCF Extraction Level 1	FC6	4096	36	FC6 (36)	90.5±2.8	88.3±7.0	92.2±4.3	89.4±6.2	91.3±5.2	80.6±5.8
	FC7	4096	98	FC7 (98)	90.0±4.4	87.7±10.2	91.7±5.1	88.8±7.4	90.9±6.2	79.6±9.4
FCF Extraction Level 2	**FC**	8192	36	FC6 (36)	90.5±2.8	88.3±7.0	92.2±4.3	89.4±6.2	91.3±5.2	80.6±5.8
Combined CF FCF Extraction	CONV_FC	19,200	25	CONV3_3 (1), CONV4_2 (3), CONV4_3 (1), CONV4_4 (1), CONV5_1 (5), CONV5_4 (14)	**94.2** ± **2.7**	**93.3** ± **5.1**	**94.9** ± **4.1**	**93.3** ± **5.6**	**94.9** ± **4.4**	**88.2** ± **5.5**

**Table 4:** The BUS image classification results obtained using the CONV feature set compared with the classification results achieved by combining the CONV feature set with handcrafted texture and morphological features. For the six performance metrics, the mean ± standard deviation values are computed across the ten folds of the cross-validation procedure performed using Dataset 1.

Features	Total No. of Features	No. of Selected Features	Selected Features	Accuracy (%)	Sensitivity (%)	Specificity (%)	PPV (%)	NPV (%)	MCC (%)
CONV feature set	11,008	25	CONV3_3 (1), CONV4_2 (3), CONV4_3 (1), CONV4_4 (1), CONV5_1 (5), CONV5_4 (14)	94.2 ± 2.7	93.3 ± 5.1	94.9 ± 4.1	93.3 ± 5.6	94.9 ± 4.4	88.2 ± 5.5
CONV feature set and texture features	11,808	25	CONV3_3 (1), CONV4_2 (3), CONV4_3 (1), CONV4_4 (1), CONV5_1 (5), CONV5_4 (14)	94.2 ± 2.7	93.3 ± 5.1	94.9 ± 4.1	93.3 ± 5.6	94.9 ± 4.4	88.2 ± 5.5
CONV feature set and morphological features	11,026	21	CONV4_4 (4), CONV5_4 (14), morphological (3)	**96.1** ± **2.2**	**95.7** ± **4.2**	**96.3** ± **3.6**	**95.1** ± **5.4**	**96.8** ± **3.1**	**92.0** ± **5.0**
CONV feature set, texture features, and morphological features	11,826	21	CONV4_4 (4), CONV5_4 (14), morphological (3)	**96.1** ± **2.2**	**95.7** ± **4.2**	**96.3** ± **3.6**	**95.1** ± **5.4**	**96.8** ± **3.1**	**92.0** ± **5.0**

**Table 5:** Performance comparison between the classification results obtained using the CONV feature set with and without features selection, the combined CONV feature set and morphological features with features selection, the handcrafted texture, morphological, and combined texture and morphological features with features selection, and the fine-tuned VGG19 model. For the six performance metrics, the mean ± standard deviation values are computed across the ten folds of the cross-validation procedure performed using Dataset 1.

Features	Total No. of Features	No. of Selected Features	Accuracy (%)	Sensitivity (%)	Specificity (%)	PPV (%)	NPV (%)	MCC (%)
CONV feature set (with features selection)	11,008	25	94.2± 2.7	93.3 ± 5.1	94.9± 4.1	93.3 ± 5.6	94.9 ± 4.4	88.2 ± 5.5
CONV feature set and morphological features (with features selection)	11,026	21	**96.1** ± **2.2**	**95.7** ± **4.2**	**96.3** ± **3.6**	**95.1** ± **5.4**	**96.8** ± **3.1**	**92.0** ± **5.0**
CONV feature set (without features selection)	11,008	-	80.5 ± 4.5	82.2 ± 9.0	79.3 ± 8.0	74.9 ± 7.1	85.6 ± 7.4	60.9 ± 8.6
Texture features (with features selection)	800	38	84.2 ± 4.3	81.0 ± 9.6	86.6 ± 6.6	82.0 ± 10.6	85.8 ± 5.5	67.7 ± 9.2
Morphological features (with features selection)	18	8	87.1 ± 4.7	82.2 ± 8.9	90.8 ± 7.9	87.0 ± 11.9	87.2 ± 6.2	73.6 ± 9.7
Texture and morphological features (with features selection)	818	29	87.9 ± 6.1	82.8 ± 12.3	91.7 ± 3.9	88.2 ± 6.1	87.7 ± 7.7	75.2 ± 12.7
Fine-tuning	-	-	88.2 ± 4.5	83.4 ± 8.0	91.7 ± 5.5	88.3 ± 8.1	88.1 ± 6.9	75.8 ± 9.1
